# Animal tumour registry of two provinces in northern Italy: incidence of spontaneous tumours in dogs and cats

**DOI:** 10.1186/1746-6148-5-39

**Published:** 2009-10-13

**Authors:** Marta Vascellari, Elisa Baioni, Giuseppe Ru, Antonio Carminato, Franco Mutinelli

**Affiliations:** 1 Histopathology Department, Istituto Zooprofilattico Sperimentale delle Venezie, Viale dell'Università 10, 35020 Legnaro (PD), Italy; 2 Epidemiology Unit, Istituto Zooprofilattico Sperimentale del Piemonte, Liguria e Valle D'Aosta, Via Bologna 148, Torino, Italy

## Abstract

**Background:**

Cancer is a major cause of death in domestic animals. Furthermore, many forms of pet neoplasm resemble that of their human counterparts in biologic behaviour, pathologic expression, and recognised risk factors.

In April 2005, a pilot project was activated so as to establish a dog and cat tumour registry living in the Venice and Vicenza provinces (Veneto Region, north-eastern Italy), with the aim of estimating the incidence of spontaneous tumours.

**Results:**

Through a telephone survey, the estimates of canine and feline populations of the catchment area turned out to be of 296,318 (CI +/- 30,201) and 214,683 (CI +/- 21,755) subjects, respectively. During the first three years, overall 2,509 canine and 494 feline cases of neoplasia were diagnosed. In dogs, the estimated annual incidence rate (IR) per 100,000 dogs for all tumours was 282 in all the catchment area, whereas in cats the IR was much lower (IR = 77). Malignant and benign tumours were equally distributed in male and female dogs, whereas cats had a 4.6-fold higher incidence of malignant tumours than benign. In both dogs and cats, purebreds had an almost 2-fold higher incidence of malignant tumours than mixed breeds. Tumour incidence increased with age in both dog and cat populations.

**Conclusion:**

This study has provided estimates of incidence of spontaneous neoplasm in companion animals. Further attempts will be made to increase the accuracy in the population size assessment and to ascertain the real gap with the official regional canine demographic registry. Veterinary practitioners may also benefit from the tumour registry insofar they may obtain data for specific breeds, age groups or geographical areas.

## Background

Cancer is an important disease in companion animals. However, developing reasonably precise estimates of cancer morbidity and mortality rates in pet animals has proven difficult to achieve. While cancer registries in human medicine have existed and evolved since the 1940s, veterinary cancer registries have existed in smaller numbers and have often been short lived and sporadic in nature, moreover, there is little up-to-date information available on the incidence of the different types of cancer in companion animals. Cancer registries serve to provide information for the evaluation of incidence and the relative risk estimates along with data for epidemiological studies. This information includes risk factor identification, treatment evaluation and location of patients for clinical trials as well as supplying cases in case-control studies. Numerous surveys and studies from extensive data collections have been performed, covering broad fields in veterinary oncology research. These studies can yield specific information but lack a population-oriented approach, and therefore, the results often cannot be extrapolated to cover the entire population. Current and prior veterinary cancer registries along with the information that they contain and their structures have been recently reviewed [1]. Few studies have attempted to estimate population-based cancer incidence rates, and the difficulties encountered in the computation of accurate companion animal population, were handled differently by each one [2-6]. One of the largest, best known and most often cited veterinary cancer registries is the California Animal Neoplasm Registry (CANR). For 3 years after 1963, data were collected from a well-defined study area, recording more than 30,000 malignant neoplastic cases on which histopathology was performed free of charge [2]. This data set has frequently been used as reference data set in the evaluation of cancer incidence in other studies and surveys as well as in textbook materials [7]. Publications derived from the CANR include estimates of cancer incidence from the reported cases in dogs and cats according to age, sex and breed, showing that skin is the most frequently affected tissue in both cats and dogs and that purebred dogs seem to be more prone to develop neoplastic diseases in all sites [3].

In Norway, a cancer registration project was initiated for canine cancer in a defined geographical region in 1990. This incidence registry includes both malignant and benign tumours and originally provided free histopathological evaluation to practitioners in the area when submitting cases for the registry [5,6].

Data from a pet insurance company was used to estimate the incidence of neoplasia in a defined population of insured dogs in the United Kingdom [5]. From a database of 130,684 insured dogs, claims relating to the investigation or treatment of tumours or tumour-like lesions during a 12-month period were accessed and followed up [5].

In 2005, a new veterinary cancer registry was established at the Royal Veterinary and Agricultural University in Denmark. Data on the resident dog population was obtained from the Danish dog registry [1]. So far, more than 1,000 submissions have been collected, and newsletters containing epidemiological overviews are published on a regular basis [4].

Recently, data from the animal tumour registry of Genoa, Italy, between 1985 and 2002 have been published [6]. The Canine Demographic Registry of the city of Genoa was used to estimate the population at risk for the computation of cancer incidence rates [6].

The aim of our study was to establish a tumour registry of dogs and cats in two provinces in the Veneto region (northern Italy). Incidence data from a three year veterinary cancer surveillance and registration are reported.

## Methods

In April 2005 the Animal Tumour Registry (ATR) of the Vicenza and Venice provinces of Veneto region (northern Italy) was established. This incidence registry includes both malignant and benign neoplasia and provides free cytological and histopathological evaluation to practitioners working in the registry's catchment area.

All veterinary practitioners in the Veneto region were informed about the registry project and invited to submit any suspected neoplasm from dogs and cats living in the Vicenza and Venice provinces. A standardized case-report form specifically designed for the collection of canine and feline tumour cases was made available to all veterinarians. The form's fields contained animal data including species, date of birth (mm/dd/yy), sex, breed, ovariohysterectomy or castration status, and place of residence. When the complete date of birth was not available, the 1^st ^day of the year was used to assign the dog/cat to the age class. Tumour data included anatomical site, size of the lesion, date of excision, clinical stadiation of the tumour and any related historical and clinical information. Formalin fixed samples were routinely processed, paraffin embedded and stained with Haematoxylin and Eosin (HE) for histological examination. Immunohistochemistry was also performed to characterise poorly differentiated neoplasm. Tumours were classified according to the World Health Organisation International histological classification of tumours of domestic animals [8] and coded according to the World Health Organization's International Classification of Disease for Oncology system (ICD-O) [9] to facilitate comparisons with existing human and animal cancer registries. Participating practitioners were also invited to report cases diagnosed by other diagnostic laboratories and cases diagnosed by ways of diagnostic imaging investigations.

In order to estimate the size of the canine population of the registry catchment area, the official Demographic Registry of the canine population established in the Veneto region was used as a primary source. Unfortunately the accuracy of the registration remains uncertain, because on one hand not all owners comply with the mandatory reporting of their pet(s) and on the other one not all dead dogs are deleted from the registry. The feline population has never been estimated and registered in the Veneto region.

To ascertain the unbiased target canine population and to estimate the feline population living in the Vicenza and Venice provinces, a telephone survey aimed at private households calculating the number of dogs and cats according to the number of people in the catchment area was made [10,11].

Given the overall number of families living within the catchment area, a random sample, stratified by municipality, of 515 private telephone numbers was obtained from the two provinces. Such a sample size would have allowed to estimate, at a 95% confidence level and with an error of 4.32%, the proportion of households owning pets (50% supposed prevalence). Telephones that were busy or not answered were recalled a minimum of four times. During the interview we asked the respondents if they had pets or not, number of pets owned, characteristics (species, sex, breed and age) and use of veterinary services.

Data about sex, breed (crossbred vs purebred), and age (4 age classes) were employed in the determination of the structure of the canine and feline populations and to obtain sex-breed-age-specific rates.

Crude and specific incidence rates (IR) were calculated as annual rate per 100,000 animals considering both the entire period and the observation period was split in two parts (18 months each). The 95% confidence intervals (CI) were constructed taking IR +/- 1,96 × SE.

## Results

**Figure 1:**
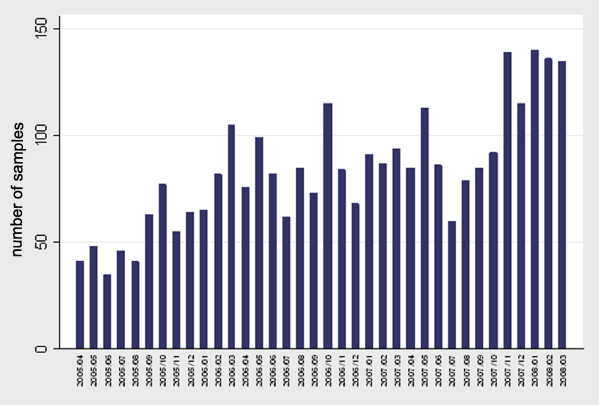
**Submission of samples across the study period (1^st ^April 2005 - 31^st ^March 2008)**.

Overall 79 veterinary clinics from the Venice province and 85 from the Vicenza province submitted samples for cytological and histological examination. Sample submission increased during the study period (Fig 1). During the past three years 2,509 pathology reports of neoplastic diseases in dogs and 494 in cats were collected. Thirty cases from dogs and four from cats were diagnosed by cytological examination. The distribution of the absolute number of tumours by species, province and malignancy is reported in table 1. In dogs, 1,269 (51%) were malignant tumours, whereas in cats 406 malignancies (82%) were diagnosed. The most frequently diagnosed neoplasms among female dogs were mammary tumours (56%), skin and soft tissue tumours (31%) and genital tract tumours (3%). Among male dogs, skin and soft tissue (56%), genital tract (13%), and the oral cavity (4%) were the most affected systems. Among cats, skin and soft tissue were the most affected sites in both males (65%) and females (49%). Detailed data are reported in table 2. Lymphomas accounted for 3% of all canine tumours.

**Table 1:** Distribution of tumours collected from the two provinces (1^st ^April 2005 - 31 March 2008)

	**DOG**		**CAT**	
	
	**VENICE**	**VICENZA**	**VENICE**	**VICENZA**
**BENIGN TUMOURS**	583	657	52	36
**MALIGNANT TUMOURS**	548	721	235	171
**TOTAL**	1131	1378	287	207

**Table 2:** Proportion of tumours by site (%)

		**DOG**			**CAT**		
		
**SITE**	**Topographic code (ICD-O)**	**Female**	**Male**	**Total**	**Female**	**Male**	**Total**
**SKIN and SOFT TISSUE**	C44; C49	30.8%	56.1%	40.8%	49.0%	65.2%	55.1%
**MAMMARY**	C50	56.4%	1.9%	34.8%	25.3%	1.1%	16.3%
**GENITAL TRACT**	C51 - C58; C60 - C63	3.2%	13.4%	7.2%	0.7%	0.6%	0.6%
**ORAL CAVITY**	C03 - C06	1.6%	4.0%	2.6%	3.0%	1.7%	2.5%
**LYMPHOID TISSUE**	C42	1.5%	3.2%	2.2%	3.3%	3.3%	3.3%
**INTESTINE**	C15 - C21	0.8%	2.8%	1.6%	2.3%	3.9%	2.9%
**LIVER**	C22	0.1%	0.5%	0.3%	1.3%	4.4%	2.5%
**LUNG**	C34	0.1%	0.2%	0.2%	2.6%	2.2%	2.5%
**URINARY TRACT**	C64 - C68	0.3%	0.2%	0.3%	1.0%	0.6%	0.8%
**OTHERS**		5.2%	17.7%	10.0%	11.5%	17.0%	13.5%
**TOTAL**		100%	100%	100%	100%	100%	100%

Of the 511 telephone numbers called, 353 complete interviews were made (Venice: n = 138; Vicenza: n = 215); 148 telephone numbers were ineligible because they resulted as being business households, or they went unanswered. Ten out of the 363 eligible residences refused to participate.

**Figure 2:**
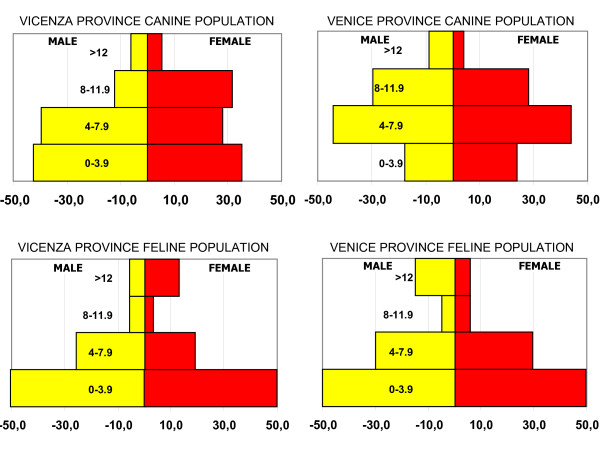
**Characteristics of canine and feline populations in the catchment area, with regard to sex distribution and the age stratification**.

The canine population reported by the Veneto's Canine Demographic Registry was equal to 162,127 in the catchment area. Based on the household telephone survey, the estimated canine population was 296,318 (CI +/- 30,201) with 153,703 (CI +/- 15,665) dogs in the Venice province and 142,615 (CI +/- 14,535) in the Vicenza province. The estimated feline population was 214,683 (CI +/- 21,755), with 97,589 (CI +/- 9,946) and 117,094 (CI +/- 11,934) subjects in the Venice and the Vicenza province, respectively. The characteristics of canine and feline populations in the catchment area, with regards to sex distribution and the age stratification, are reported in Fig 2.

To calculate the incidence rates, all denominators used come from the household survey. Considering the size of population to be constant, the annual IR has been calculated as the number of cases/100,000 dogs or cats.

In dogs, the estimated crude annual incidence rate for all tumours per 100,000 dogs (IR) was 282.2 in all the catchment area. In dogs, the IR of malignant tumours was 143 (CI: 130, 159) vs. 140 (CI: 127, 155) of benign tumours. In females the IR of malignant and benign tumours was 166 (CI: 151, 185) and 153 (CI: 139, 171) respectively, while in males it was 101 (CI: 92, 113) and 109 (CI: 99, 122) respectively. In purebred dogs the IR for malignant tumours was two folds higher than in crossbred dogs (183, CI: 166, 204 vs. 89, CI: 80, 99 respectively).

In cats, the crude IR was 77 (CI: 70, 85) in all the catchment area. The IR of malignant and benign tumours was 63 (CI: 57, 70) and 14 (CI: 12, 15) respectively. In female cats the IR of malignant tumours was 70 (CI: 64, 78) vs. 17 (CI: 15, 19) of benign tumours. In males, the IR of malignant and benign tumours was 52 (CI: 47, 57) and 10 (CI: 9, 11), respectively. The IR of malignant tumours was 168 (CI: 153, 187) in pure breed cats and 85 (CI: 77, 94) in cross breeds, while IRs of benign tumours were comparable in pure and cross breed groups. In table 3 the crude IRs for the first (0 - 18 months) and the second (19 - 36 months) period of registration are reported.

**Table 3:** Crude period-specific IRs and their 95% confidence intervals (CI)

	**0 - 18 months**			**19-36 months**		
	
**DOG**	**CASES**	**IR**	**CI**	**CASES**	**IR**	**CI**
MALIGNANT TUMOURS	550	124	112-138	719	162	147-180
BENIGN TUMOURS	436	98	89-109	804	181	164-201
**CAT**						
MALIGNANT TUMOURS	184	57	52-64	222	69	63-77
BENIGN TUMOURS	29	9	8-10	59	18	17-20

**Figure 3:**
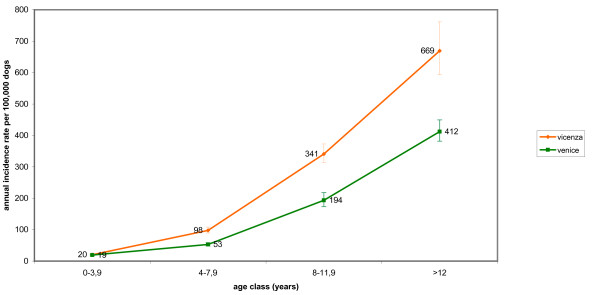
**Age-specific incidence rates for malignant tumours in dogs in the two provinces**.

**Figure 4:**
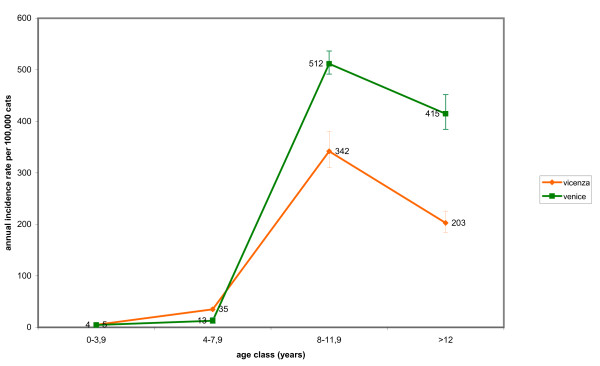
**Age-specific incidence rates for malignant tumours in cats in the two provinces**.

**Figure 5:**
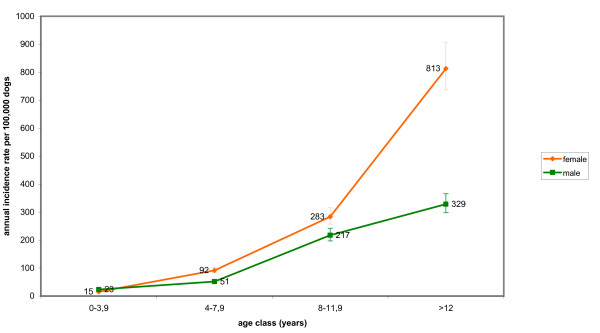
**Age and sex-specific incidence rates for malignant tumours in dogs**.

**Figure 6:**
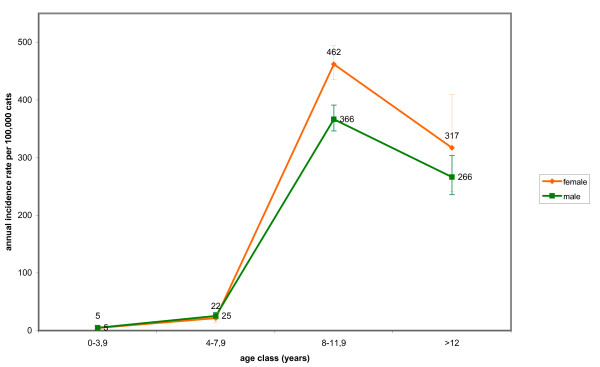
**Age and sex-specific incidence rates for malignant tumours in cats**.

The IR of lymphoma in dogs reached maximum values at the age of 12 years and no significant difference between sexes was observed. Purebred dogs had a significant higher risk (2.3 times) than crossbred dogs of developing malignant tumours of lymphoid tissues. Age-specific IRs for malignant tumours in dogs and cats in the two provinces (Fig. 3, 4) and by sex (Fig. 5, 6) are provided.

## Discussion

This study allowed (i) estimation of the canine and feline population in two provinces of Veneto region (northern Italy), (ii) characterization of the population and (iii) estimation of spontaneous neoplasm incidence in dogs and cats living in the catchment areas.

To estimate the owned-pet dog and cat populations in the Vicenza and Venice provinces, we carried out a household telephone survey. The random-digit dial method is widely used and has been proven to be valid and reliable for many purposes [12]. Notwithstanding, people may not be listed in the phone book directory and may have a mobile phone only. As a consequence the sample selected based on a random sample of telephone numbers as well as individual habits, e.g. the time spent at home, could have introduced some bias. However, telephone lines that were busy or not answered were recalled a minimum of 4 times on different days and hours.

No previous data were available about the cat population in the Veneto region. The estimated dog population resulted to be higher than that reported in the demographic registry. Despite the fact that the registration of dogs is a legal requirement the combined efforts of both the official veterinary services and the veterinary practitioners, seem to be insufficient to assure a complete and unbiased dog registration. Availability of accurate estimates of pet populations is essential to conduct any population based epidemiological study, to evaluate the incidence of infectious and neoplastic diseases, to highlight geographical differences providing insight into the aetiology of the diseases and the possible environmental risk factors related.

In fact, it has been claimed that cancer occurrence in pets may warn of possible environmental causes of cancers in humans for specific geographic areas. Several noteworthy examples of environmental exposures that increase risk of cancer in companion animals have been e.g. a significant association with urban air pollutants was noted for dogs with tonsillar carcinoma [13]; bladder cancer in dogs may be associated with exposures to environmental contaminants, particularly herbicide and insecticide products, and some of these agents may also be associated with human cancer risk [14].

In our survey, the most frequently diagnosed tumours in dogs affected the mammary gland, skin and soft tissue, genital tract and oral cavity. These findings are in accordance with those reported by the animal tumour registry of Genoa (Italy) [6], and by the Norway canine cancer registry [15]. Cancers of the skin and mammary gland were by far the most common type of tumour encountered in these registries, followed by oral and testicular cancers [6,15]. Mammary gland, genital, and skin tumours are easier to recognize by physical examination, as opposed to other tumours affecting internal organs that require specific investigations such as X-ray, computed tomography scanning (CT), magnetic resonance imaging (MRI) and ultrasound examination. In our registry, cases diagnosed by cytology and diagnostic imaging were also included, to minimise the possible underestimation of tumours affecting internal organs. Notwithstanding, due to the fact that CT and MRI are not routinely used as diagnostic tools, and post mortem investigations are not always required by owners, IRs are likely to have been underestimated.

In our registry, lymphomas rise particular concern, accounting for 3% of all canine tumours, and the IR increased constantly with age and in purebred dogs. These findings are in agreement with data from the Alameda Registry (1968) [3] and with the age specific incidence reported by Edwards and coll. (2003) in the UK population of insured dogs [16]. In the Genoa registry, non-Hodgkin's lymphoma was one of the most frequently diagnosed tumours, particularly in male dogs (20%) [6], and showed the highest IR in the age group of > 7-9 years dogs, decreasing in older dogs.

The estimated IR for all cancers in dogs was 143 in all the catchment area. Published IRs for approximately all cancers ranged between 310 and 958 per 100,000 dogs [6]. In the canine Genoa registry, IRs for all cancers increased significantly in the calendar period 2000-2002 compared with the period 1985-1989 showing a monotonic increase across the entire study period [6]. Considering the difference between the crude IRs of the two periods of our study (table 3) a possible underestimation of our rates can be hypothesized, due to the relatively short life of this registry and the increasing trend of sample submission observed during the registration period. Continuing sample collection may provide more stable and refined incidence data. Furthermore, differences in the estimated IRs between registries, could be attributable to the different methods applied to estimate the population, as well as to the completeness of the reported cases.

IRs of malignant tumours in dogs increased with age, peaking at age > 12 years. A similar pattern was observed in the canine Genoa registry, with a peak of IRs in dogs > 9-11 years [6].

In our survey, purebred dogs and cats seem to have a higher risk to develop malignant tumours than crossbred. Notwithstanding, it is noteworthy the wide breed-variation because of the genetic diversity between breeds. More data are necessary to make further investigation about IRs in different purebreds. Preliminary data from the Danish dog registry show increased relative risk in breeds such as Bull terrier, American Bull dogs and Boxers and the most frequently occurring neoplasm is shown to be mammary tumours [1].

It is difficult to make a direct comparison between our and other cancer registries because of differences in the estimated at risk populations, inclusion criteria and tumour diagnosis and classification. Within the veterinary community, the agreement to use a specific international classification system is strongly desirable, in order to increase the comparability of the information gathered in the existing veterinary cancer registries.

## Conclusion

In conclusion, this study has allowed an estimation and characterization of the canine and feline population living within two provinces of the Veneto region (northern Italy), and has provided estimates of incidence of spontaneous neoplasm in companion animals. Further attempts will be made to increase the accuracy in the population size assessment and to ascertain the real gap with the official regional canine demographic registry.

Continuation of the registration and increasing the database may allow evaluation of temporal trends and potential environmental and individual risk factors, such as age and breed, which could affect susceptibility to a specific cancer. Veterinary practitioners may benefit from the tumour registry by obtaining data for specific breeds, age groups or geographical areas.

## Authors' contributions

MV led the project, made histological diagnosis and drafted the manuscript; EB managed estimation of the canine and feline population, and incidence data; GR supervised the epidemiological approach of the study, and contributed to the manuscript; AC made cytological and histological diagnosis; FM supervised the project and contributed to the manuscript.
